# A New Optimization Strategy of Highly Branched Poly(β-Amino Ester) for Enhanced Gene Delivery: Removal of Small Molecular Weight Components

**DOI:** 10.3390/polym15061518

**Published:** 2023-03-18

**Authors:** Yinghao Li, Xianqing Wang, Zhonglei He, Zishan Li, Melissa Johnson, Bei Qiu, Rijian Song, Sigen A, Irene Lara-Sáez, Jing Lyu, Wenxin Wang

**Affiliations:** Charles Institute of Dermatology, School of Medicine, University College Dublin, D04V1W8 Dublin, Ireland

**Keywords:** gene transfection, highly branched poly(*β*-amino ester), non-viral vector, polymer component, pDNA delivery, step-wise precipitation

## Abstract

Highly branched poly(β-amino ester) (HPAE) has become one of the most promising non-viral gene delivery vector candidates. When compared to other gene delivery vectors, HPAE has a broad molecular weight distribution (MWD). Despite significant efforts to optimize HPAE targeting enhanced gene delivery, the effect of different molecular weight (MW) components on transfection has rarely been studied. In this work, a new structural optimization strategy was proposed targeting enhanced HPAE gene transfection. A series of HPAE with different MW components was obtained through a stepwise precipitation approach and applied to plasmid DNA delivery. It was demonstrated that the removal of small MW components from the original HPAE structure could significantly enhance its transfection performance (e.g., GFP expression increased 7 folds at *w/w* of 10/1). The universality of this strategy was proven by extending it to varying HPAE systems with different MWs and different branching degrees, where the transfection performance exhibited an even magnitude enhancement after removing small MW portions. This work opened a new avenue for developing high-efficiency HPAE gene delivery vectors and provided new insights into the understanding of the HPAE structure–property relationship, which would facilitate the translation of HPAEs in gene therapy clinical applications.

## 1. Introduction

Gene therapy has become an essential field in medicine due to increased demand for treatments to treat rare and genetic diseases as well as the rising prevalence of cancer. The gene therapy market is expected to grow exponentially in the coming years as technology advances, and its potential applications are becoming more widely known. The global gene therapy market, which was valued at USD 6.5 billion in 2020, is projected to grow at a compound annual growth rate of 20.2% to reach USD 23.7 billion by 2027 [1]. However, the lack of safe and efficient gene delivery vectors continues to hinder the translation of gene therapy treatments to large-scale clinical applications [2,3].

In the context of the recent pandemic, the extensive use of lipoplexes in the preparation of mRNA vaccines has brought further attention to the use of non-viral vectors for gene delivery. While viral vectors have traditionally been the preferred choice for gene carriers due to their high trans-gene expression, safety concerns such as immunogenicity and broad tropism have limited their application [4,5]. As a result, scientists have been exploring alternative gene carriers including cationic polymers and cationic lipids [6]. Liposome-based vectors, in particular, have gained traction due to their ability to effectively deliver mRNA and other genetic materials. However, lipoplexes suffer from limitations such as poor reproducibility and high cytotoxicity in certain cell types as well as the potential for inflammatory responses [7,8,9]. In comparison, polymer vectors offer several advantages including high cargo capacity, biosafety, ease of operation, and modification [10,11,12,13].

After decades of effort, a large number of polymer vectors have been developed [6,10,14,15]. Among them, poly(β-amino ester)s (PAEs) have emerged as a significant class of highly effective gene delivery vectors. In 2000, linear PAEs (LPAEs) were first designed by Langer et al. [16], and several high-performance LPAEs have since been developed for gene delivery both in vitro and in vivo [17,18,19,20,21,22,23,24,25]. Furthermore, in 2016, Wang et al. constructed highly-branched PAEs (HPAEs) via a facile “A2 + B3 + C2” Michael addition strategy [26]. The gene transfection efficiency of optimized HPAEs mediated up to multitude-fold enhancement in comparison to the corresponding LPAEs. Since then, continuous efforts have been made to optimize the structure of HPAE (e.g., molecular weight, branch ratio, and terminal groups, etc.) in order to improve its gene transfection performance. However, despite years of research, only minor improvements in HPAE transfection efficiency have been achieved [27,28,29].

Understanding the unique structural characteristics that distinguish HPAEs from other vectors is critical for breaking down this bottleneck and facilitating the development of high-efficiency HPAE gene delivery vectors. Unlike viruses and lipids, which have defined chemical structures, HPAE, synthesized by step-growth polymerization (SGP), has a broad molecular weight distribution (MWD) (dispersity (*Ɖ*) > 2), which is a mixture of components with different molecular weights (MWs) [26]. However, previous reports have proven that low MW HPAEs are not conducive to transfection, with transfection efficiency increasing as PAE MWs increase [28,30,31]. Inspired by this, the existence of abundant small MW components (even after purification) in HPAE polymers might compromise their overall transfection capability. Conversely, an improved gene delivery performance could be obtained by removing the small MW components from HPAE vectors.

Based on the above hypothesis, in this work, a stepwise precipitation method was used to remove the small MW components in HPAEs. A series of HPAEs with different MW components were achieved, and their transfection behavior was investigated in vitro. By comparing the transfection results from HPAEs with different polymer components, the above hypothesized optimization strategy was proven, whereby eliminating the small MW components resulted in the development of a highly efficient HPAE gene delivery vector with transfection capability that surpassed the well-known commercial reagent jetPEI. This optimization strategy was further validated in HPAEs with different MWs and branching degrees (BDs). 

## 2. Materials and Methods

### 2.1. Materials

1,4-Butanediol diacrylate (BDA), 5-amino-1-pentanol (S5), and pentaerythritol tetraacrylate (PTTA) were purchased from Sigma-Aldrich. 1-(3-Aminopropyl)-4-methylpiperazine (E7) was purchased from Fisher Scientific. Lithium bromide (LiBr) for GPC measurements was purchased from Sigma-Aldrich. Dimethyl sulfoxide (DMSO), dimethylformamide (DMF), acetone, and diethyl ether were purchased from Fisher Scientific. Deuterated chloroform (CDCl_3_) and tris acetate-EDTA (TE) buffer were purchased from Sigma-Aldrich. Hank’s balanced salt solution and Alamar Blue Assay Kit were purchased from Sigma and Invitrogen. Dulbecco’s phosphate buffered saline (PBS) and PicoGreen assay kits were purchased from Life Technologies and used as per the manufacturer’s protocol. Sodium acetate (Sigma) was diluted to 0.025 M before use. Cell culture Dulbecco’s modified Eagle medium (DMEM) was purchased from Sigma. Fetal bovine serum (FBS) purchased from Gibco was filtered through 0.2 µm filters before use. The commercial green fluorescent protein plasmid (gWiz-GFP) was obtained from Aldevron, Fargo, ND, USA. JetPEI was purchased from Polyplus Transfection, Illkirch-Graffenstaden, Strasbourg, France.

### 2.2. Polymer Synthesis

Highly branched PAEs (HPAEs) were synthesized through a facile Michael addition reaction. The monomer feeding ratios and reaction conditions are listed in Table S1. In order to synthesize HPAE-A1, BDA (3.96 g), S5 (2.06 g), and PTTA (0.70 g) were dissolved in DMSO (1.53 mL). Then, the solution was bubbled under argon for 15 min to remove oxygen. Afterward, the reaction mixture was merged into the preheated oil bath and reacted with stirring at 90 °C. Agilent 1260 Infinite gel permeation chromatography (GPC) and nuclear magnetic resonance (NMR) were used to monitor the reaction. The reaction was stopped by diluting the mixture to 100 mg/mL with DMSO when *M*_w,GPC_ approached desired value. E7 (2.51 g) was then added to endcap the acrylate-terminated base polymer at room temperature for 48 h. After that, HPAE polymers were precipitated into diethyl ether for purification and dried under vacuum before being stored at −20 °C. 

### 2.3. Stepwise Precipitation

After achieving HPAE polymers, they were applied to stepwise precipitation to obtain HPAEs with different polymer component combinations. Taking HPAE-A1 as an example, the stepwise precipitation procedure is as follows: HPAE-A1 was dissolved in acetone at a concentration of 100 mg/mL, then the solution was slowly added into the mixed solvent of acetone and diethyl ether (*v/v* = 1/9) under gentle agitation at room temperature. The precipitate was collected as HPAE-A2. Then, HPAE-A2 was redissolved in acetone and precipitated into another mixed solvent with a higher acetone extent (acetone/diethyl ether = 2/8) to generate the component HPAE-A3. By repeating the step-by-step precipitation process, HPAE-A2 to A4 were obtained. HPAE-B3 to HPAE-E3 were obtained following the same procedure.

### 2.4. Molecular Weight and Dispersity Measurements

The number average molecular weight (*M*_n,GPC_), weight average molecular weight (*M*_w,GPC_), and dispersity (*Ɖ*) of the HPAE polymers were determined by GPC equipped with a refractive index detector (RI), a viscometer detector (VS DP), and a dual angle light scattering detector (LS 15° and LS 90°). To monitor the molecular weight of polymers during the polymerization process, 20 µL of the reaction mixture was collected at different time points, diluted with 1 mL of DMF, filtered through a 0.2 µm filter, and then measured by GPC. The columns (PolarGel-M, Edinburgh, UK, 7.5 mm × 300 mm, two in series) were eluted with DMF and 0.1% LiBr at a flow rate of 1 mL/min at 60 °C. Columns were calibrated with linear poly(methyl methacrylate) (PMMA) standards.

### 2.5. Proton Nuclear Magnetic Resonance (^1^H NMR)

The chemical structure, branching degree, and composition of the HPAE polymers were measured by ^1^H NMR. The 10 mg polymer samples were dissolved in 800 µL CDCl_3_. Measurements were carried out on a Varian Inova 400 MHz spectrometer.

### 2.6. Cell Culture

Recessive dystrophic epidermolysis bullosa keratinocytes cells (RDEBK) were cultured using standard cell culture techniques in keratinocyte growth complete FAD medium (KCa). Human embryonic 293 kidney cells (HEK293) were cultured in Dulbecco’s modified Eagle medium high glucose containing 10% fetal bovine serum and 1% penicillin-streptomycin. Cells were cultured at 37 °C with 5% CO_2_ in a humid incubator under standard cell culture techniques.

### 2.7. Polyplex Preparation

Generally, the polymers were initially dissolved in DMSO to stock solutions (100 mg/mL), and then the stock solutions were further diluted with 25 mM sodium acetate buffer according to the *w/w* ratio. DNA was diluted to 0.1 mg/mL with sodium acetate buffer. The polymer solutions were added into the DNA solution, vortexed for 10 s, and allowed to stand for 15 min.

### 2.8. DNA Condensation Measurement (Agarose Gel Electrophoresis)

Agarose gel electrophoresis was used to determine the DNA condensation ability of HPAEs. A total of 0.5 μg of DNA was used for each sample. Polyplexes with a series of *w/w* ratios were prepared as above. After that, 10 μL of the polyplex solution was loaded into the wells in the agarose gel, and naked DNA was used as the control. Gel electrophoresis was performed at 120 V for 40 min and the images were captured using Syngene’s G:BOX.

### 2.9. DNA Binding Affinity (PicoGreen Assay)

PicoGreen assays were used to measure the DNA binding efficiency of polymers. A total of 0.5 μg of DNA was used for each sample. Polyplexes were prepared as described above under different *w/w* ratios. Afterward, 10 μL of PicoGreen working solution (prepared by diluting 4 μL of PicoGreen with 800 μL of TE buffer) was added and left to incubate for 5 min. Then, 100 μL of pure water was added to a black 96-well plate, followed by 20 μL of polyplex/PicoGreen solution. Fluorescence was measured using a SpectraMax M3 plate reader equipped with an excitation at 490 nm and an emission at 535 nm.

### 2.10. Polyplex Size and Zeta Potential Measurement

Polyplex sizes and zeta potentials were measured with a Malvern Instruments Zetasizer (Nano-2590) at a scattering angle of 173°. Polyplexes were prepared as above described under different *w/w* ratios. For size measurement, polyplex was diluted in 1 mL media with 10% FBS. For zeta potential measurement, polyplex was diluted in 1 mL of 25 mM sodium acetate. Polyplex sizes and zeta potentials were measured a minimum of three times at 25 °C.

### 2.11. Transfection Experiments

GFP reporter gene transfection was first performed to evaluate the gene transfection efficiency of HPAEs and screen out the best-performing candidate. RDEBKs and HEKs were seeded in 96-well plates. The next day, 0.5 μg of plasmid DNA encoding GFP was used for each well. Polyplexes were prepared at polymer/DNA *w/w* ratios of 10:1, 20:1, and 30:1 in 10 μL of sodium acetate per well, mixed with 90 μL of fresh culture medium as the transfection medium. GFP expression of cells was visualized under a fluorescence microscope (Olympus IX81, Dublin, Ireland) 48 h post-transfection. The intensity of GFP fluorescence was then analyzed and semi-quantified using ImageJ software (NIH, Bethesda, Rockville, MD, USA). After imaging, the cell viability of the treated cells was measured using the Alamar Blue Kit according to the instruction manual.

### 2.12. Cytotoxicity Assessment (Alamar Blue Assay)

To perform the Alamar Blue assay, the cell supernatants were first removed, then the cells were washed with PBS or Hanks buffer, followed by the addition of 10% Alamar Blue reagent in the solution. Living, proliferating cells maintain a reducing environment within the cytosol of the cell, converting the non-fluorescent ingredient resazurin in Alamar Blue to the highly fluorescent compound resorufin. This reduction results in a color change from blue to light red. It allows for the quantitative measurement of cell viability based on the increase in overall fluorescence and color of the media. The Alamar Blue solution from each well was transferred to a fresh flat-bottomed 96-well plate for fluorescence measurements at 590 nm. Control cells without any treatment were used to normalize the fluorescence values and plotted as 100% viable.

## 3. Results and Discussion

### 3.1. Synthesis and Characterization of HPAEs with Different Polymer Components

**Scheme 1:**
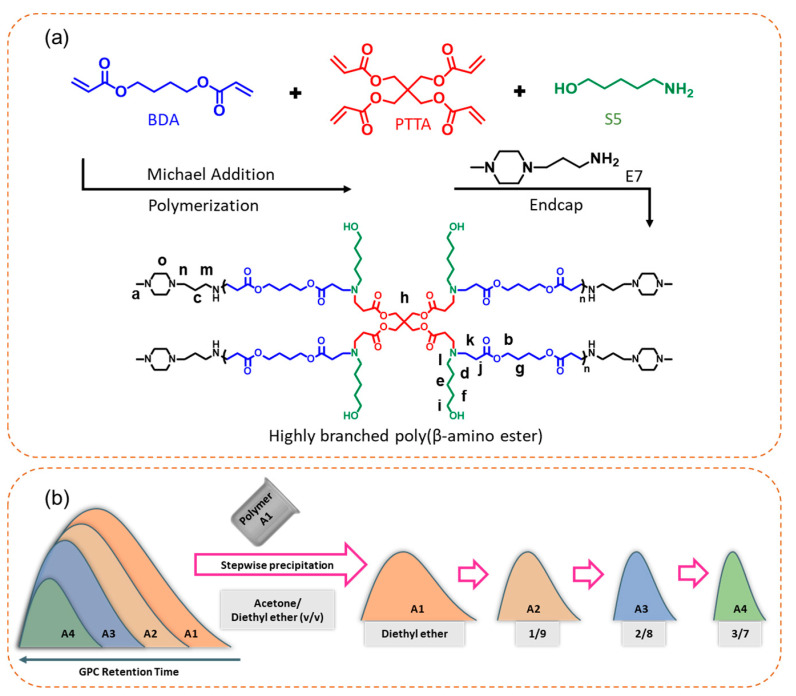
Schematic illustration of the HPAE synthesis and precipitation processes. (**a**) HPAE-A1 was synthesized via the Michael addition approach from 1,4-butanediol diacrylate (BDA), pentaerythritol tetraacrylate (PTTA), 5-amino-1-pentanol (S5), and 1-(3-aminopropyl)-4-methylpiperazine (E7, endcapping reagent). The molar ratio of BDA to PTTA to S5 is approximately 10:1:10. (**b**) HPAE-A1 was precipitated into HPAE-A2 to A4, respectively, by precipitating into a solvent mixture of acetone/diethyl ether (*v/v* = 1/9 to 3/7). HPAE-A2 to A4 were collected from the precipitates.

**Figure 1:**
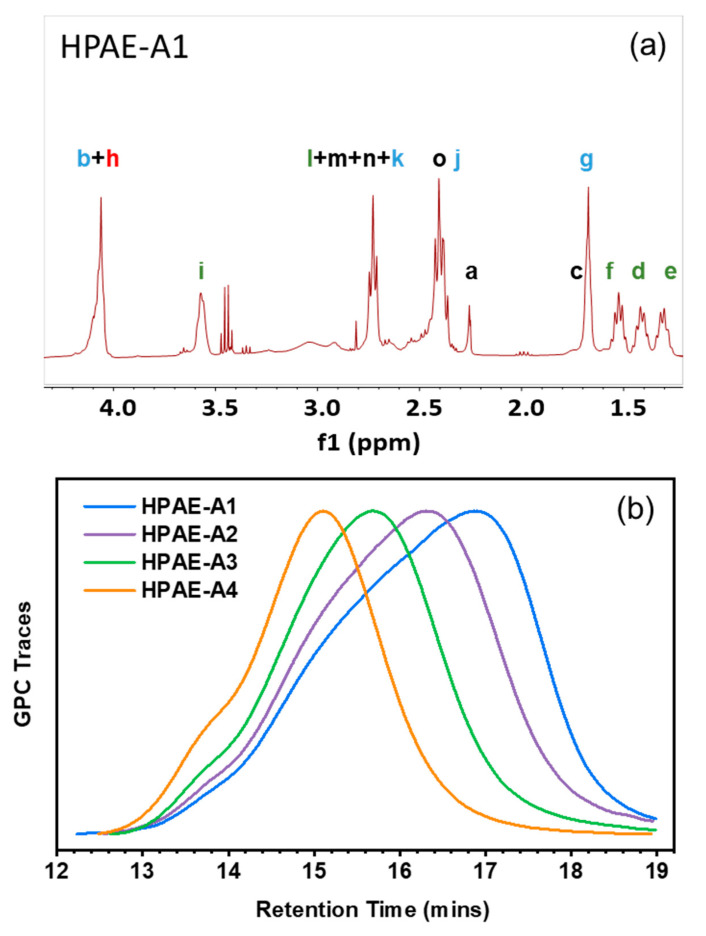
Polymer structure characterization. (**a**) ^1^H NMR spectrum of HPAE-A1. Branching degree of HPAE-A1 is 0.12, which is calculated from the equation: molar ratio of PTTA/BDA = [(*I*_g+h_–*I*_b_)/8]/[*I*_b_/4], where *I*_g+h_, and *I*_b_ stand for the integral intensity of peak g+h and peak b in the ^1^H NMR spectrum. The hydrogen corresponding to the letter can be found in the Scheme 1. The black letters represent E7, green letters represent S5, blue letters represent BDA, and red letter represents PTTA. (**b**) GPC traces of HPAE-A1 to A4.

Previously, HPAEs were obtained by direct precipitation into diethyl ether after synthesis [26]. However, only monomers and oligomers can be removed *via* direct precipitation, leaving a large amount of small MW components in the polymer mixture, potentially compromising the gene transfection performance of HPAEs [28,30,31]. Here, we propose a stepwise precipitation strategy to optimize the polymer component combinations in HPAEs to enhance their gene delivery efficiency. As illustrated in Scheme 1a, HPAE-A1 was first synthesized via a typical “A2 + B4 + C2” Michael addition approach using well-studied BDA, PTTA, and S5 as backbone monomers. A tertiary amine E7 was further added to endcap the synthesized polymers (Figure S1) [26]. Subsequently, as depicted in Scheme 1b, the end capped HPAE mixture was precipitated into diethyl ether to generate HPAE-A1. The chemical structure of HPAE-A1 was characterized by GPC and ^1^H NMR (Figure 1, and entry 1 in Table 1). Then, HPAE-A1 was redissolved in acetone and precipitated into a solvent mixture of acetone/diethyl ether (*v/v* = 1/9). The precipitate was then collected as HPAE-A2. By repeating this stepwise precipitation process in solvent mixtures with increasing acetone content (acetone/diethyl ether = 2/8 to 3/7), HPAE-A3 and HPAE-A4 were obtained, respectively. Figure 1 and entries 1–4 of Table 1 show the GPC characterization results of HPAE-A1 to A4. By removing the small MW components of varying degrees from HPAE-A1, a gradual movement of the MWs of HPAE-A1 to A4 from low to high (*M*_w,GPC_ = 15.0 to 34.9 kDa) could be clearly observed.

### 3.2. Evaluation of the Gene Transfection Performance of HPAE-A1 to A4

**Figure 2:**
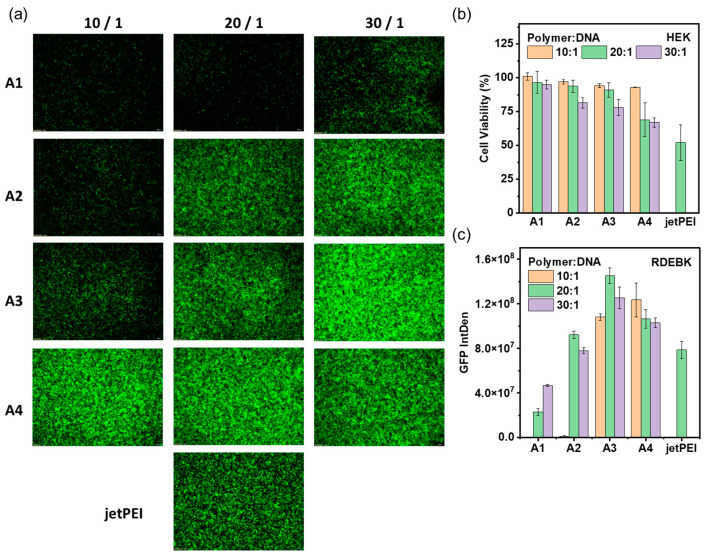
Comparison of gene transfection efficiency and cytotoxicity of HPAEs with different polymer component combinations. (**a**) Microscopic images of HEK cells 48 h post transfection (*w/w* = 10:1 to 30:1). (**b**) Cell viability of HEK cells 48 h post transfection. (**c**) GFP expression of RDEBK cells treated with polyplexes formulated with HPAEs. The commercial reagent (jetPEI) was used for comparison.

Based on the above synthesized HPAE-A1 to A4, to validate whether the optimization of the polymer component combination in HPAE (by the removal of small MW components) can enhance their gene transfection performance, the transfection behavior of HPAE-A1 to A4 was evaluated at different polymer/DNA ratios in common human-derived HEK-293 cells and a genetic disease model of RDEBK cells, respectively. Impressively, compared to HPAE-A1, with the removal of small MW components, the GFP expression of HPAE-A2 and HPAE-A3 in HEK cells was significantly enhanced (Figure 2a) with high cell viability maintained (>75%, Figure 2b). For HPAE-A4, its transfection efficiency slightly decreased compared to HPAE-A3 at the polymer/DNA weight ratio of 30/1 due to the increased cytotoxicity (Figure 2b). The same tendency was also observed in RDEBK cells post-transfection (Figure 2c and Figure S2), where HPAE-A3 exhibited better transfection performance, also far surpassing the well-known commercial reagent jetPEI. These results demonstrate that removing small MW components from HPAEs can significantly enhance transfection performance. Meanwhile, in terms of both high transfection efficiency and low cytotoxicity, HPAE-A3—generated by the three-step precipitation—is the most favorable for HEK and RDEBK gene transfection.

### 3.3. Mechanism Discussion of the Enhanced Gene Transfection Performance

**Figure 3:**
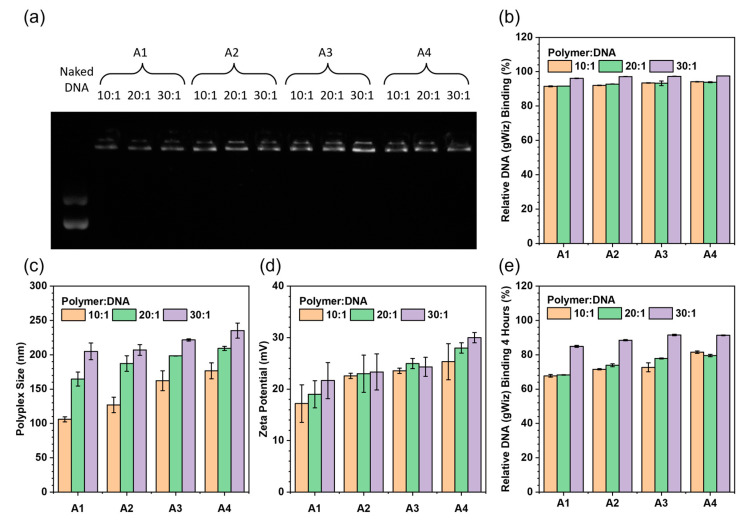
Comparison of the physicochemical characteristics of polyplexes based on HPAE-A1 to A4. (**a**) Agarose gel electrophoresis shows that HPAE-A1 to A4 all have strong interaction with DNA, which retarded the DNA effectively. (**b**) Assessment of the DNA binding capability of HPAE-A1 to A4 vectors at various polymer/DNA weight ratios (*w/w*) using the PicoGreen assay. (**c**) Polyplex sizes measured by DLS at different *w/w* ratios. (**d**) Polyplex zeta potential measurement. (**e**) DNA protection capability of HPAE-A1 to A4 under acid conditions, evaluated by the DNA binding efficiencies after 4 h incubation at 37 °C using the PicoGreen assay.

To understand the underlying mechanism behind the enhancement of gene transfection after eliminating the small MW components within HPAE, several vital factors that determine the polymer gene delivery performance including DNA condensation, DNA binding, polyplex size, zeta potential, and DNA protection capability were systematically investigated. The DNA condensation ability of HPAE with different polymer components (HPAE-A1 to A4) was first determined by agarose gel electrophoresis. As shown in Figure 3a, for all HPAEs, no DNA shifting bands were observed, indicating that HPAE-A1 to HPAE-A4 all retarded the DNA effectively. Then, the binding affinity between DNA and HPAE-A1 to A4 was quantified with a PicoGreen assay. According to Figure 3b, all HPAEs exhibited over 90% DNA binding over the range of tested polymer/DNA weight ratios (*w/w*, from 10:1 to 30:1). The DNA binding affinity only slightly increased from HPAE-A1 to HPAE-A4 (91% for HPAE-A1 and 94% for HPAE-A4 at *w/w* = 10:1). These results demonstrate that all four positively charged HPAEs can shield and bind with the negatively charged DNA effectively, thus this step should not be considered as the cause for the distinct transfection efficacy shown in Figure 2.

Furthermore, for successful gene delivery, HPAE vectors must be able to package DNA to form nano-sized polyplexes. The polyplex sizes based on HPAE-A1 to A4 were measured using dynamic light scattering (DLS). As displayed in Figure 3c, in serum-containing media at all tested *w/w* ratios from 10/1 to 30/1, HPAE-A1 to A4 could effectively condense DNA into polyplexes with sizes less than 250 nm, indicating that they are good candidates for entering cells via clathrin-mediated endocytosis [32]. However, the polyplex sizes clearly increased from HPAE-A1 to HPAE-A4 (from 106 nm to 176 nm at different *w/w* ratios = 10:1). This result indicates that with the removal of small MW components to varying degrees, more high MW components might be involved in polyplex formation, thus offering stronger DNA protection from degradation. In terms of DNA protection, especially in acidic endosomes, it is one of the key factors required for successful gene transfection. The enhanced DNA protection capability of HPAE-A2 to A4 (by removing small MW components from HPAE-A1) is reflected in Figure 3e. In Figure 3e, the DNA binding efficiencies of polyplexes based on HPAE-A1 to A4 were evaluated under an acid condition (in 25 mM sodium acetate) after 4 h incubation at 37 °C. As predicted above, HPAE-A3 and HPAE-A4, which have bigger polyplex sizes and fewer small MW components, exhibited better DNA protection capabilities (i.e., maintained higher DNA binding efficiencies after 4 h of incubation under acid conditions). The zeta potential of the HPAE/DNA polyplexes was also examined, since the positive surface charges can facilitate particle cellular uptake [6,33]. As can be seen in Figure 3d, for a given *w/w* ratio, polyplexes formed by HPAEs with fewer small MW components exhibited higher zeta potential. This means that, when compared to HPAE-A1, HPAE-A2 to HPAE-A4 have a more positively charged surface, which could potentially enhance their cellular uptake. The above mechanistic research outcomes demonstrate that by eliminating small MW components from HPAE vectors, their corresponding polyplexes can hold a higher surface potential and optimized polyplex internal structure, which ultimately enhances their gene transfection efficiency synergistically.

### 3.4. Application—Enhanced Gene Delivery of HPAEs with Different MWs and Branching Degrees (BDs)

**Figure 4:**
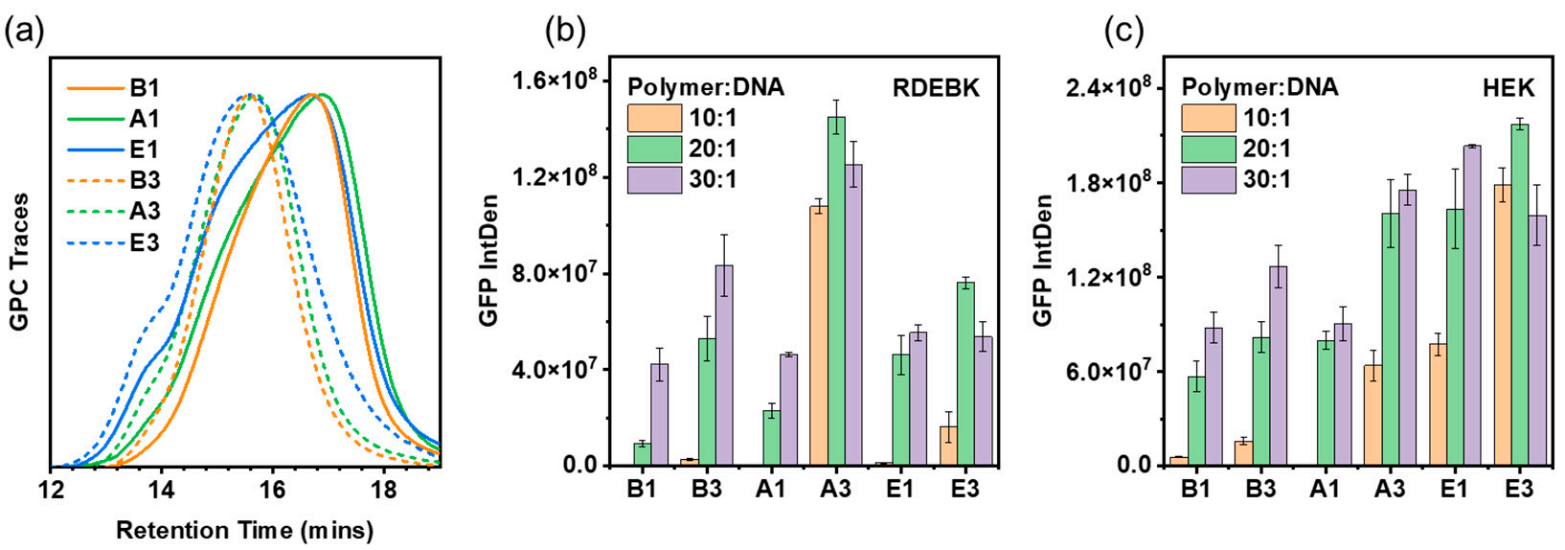
GPC characterization and in vitro assessment to investigate the applicability of removing small MW components in HPAEs with different molecular weights (HPAE-A1, A3, HPAE-B1, B3, and HPAE-E1, E3). (**a**) GPC traces of HPAE-B1, HPAE-A1, HPAE-E1, and their optimized products HPAE-B3, HPAE-A3, HPAE-E3; transfection efficiency of polyplexes formulated with HPAE-A1, A3, HPAE-B1, B3, and HPAE-E1, E3 at different *w/w* ratios 48 h post-treatment: GFP expression of (**b**) RDEBK and (**c**) HEK cells treated with HPAE/DNA polyplexes.

Furthermore, to demonstrate the universality of the above HPAE component optimization strategy in varied types of HPAE vectors, its applicability was assessed by extending to HPAEs with different MWs and BDs. First, HPAE-B1 and HPAE-E1, which have similar chemical compositions to HPAE-A1 but different *M*_w,GPC_ (*M*_w,GPC_ of HPAE-A1 = 15.0 kDa, *M*_w,GPC_ of HPAE-B1 = 12.6 kDa, *M*_w,GPC_ of HPAE-E1 = 20.1 kDa), were prepared by simply changing the polymerization time of these base polymers (entry 5 and entry 11 in Table 1, Figure 1a, Figure 4a, Figures S3–S5 and S11). Afterward, based on the study in Section 3.2, the most optimal three-step precipitation procedure was applied to stepwise precipitate HPAE-B1 and HPAE-E1 to generate HPAE-B3 and HPAE-E3 with a *M*_w,GPC_ of 21.0 kDa and 28.3 kDa, respectively (Figure 4a, entry 6 and entry 12 in Table 1). Then, HPAE-B1, HPAE-A1, HPAE-E1, and their corresponding optimized products HPAE-B3, HPAE-A3, and HPAE-E3 were applied to transfect RDEBK and HEK cells using green fluorescent protein (GFP)-encoding DNA as the reporter gene. 

Figure 4b,c shows similar results for these three HPAE groups, namely, that the GFP expression of all optimized HPAEs (HPAE-A3, HPAE-B3, and HPAE-E3) increased compared to their original products (HPAE-A1, HPAE-B1, HPAE-E1) in both RDEBK and HEK cells. Furthermore, transfection results with RDEBK cells (Figure 4b) showed that the transfection efficiency of the optimized HPAE-A3 with *M*_w,GPC_ of 23.6 kDa was enhanced threefold than that of HPAE-A1, with a preserved high cell viability of 85% (Figure S6), exhibiting the most robust GFP expression among all of the tested HPAEs. However, for HEK cell transfection (Figure 4c), HPAE-E1 and HPAE-E3 with higher *M*_w,GPC_ exhibited higher transfection capabilities than HPAEs with lower *M*_w,GPC_ including their optimized products (HPAE-B1, B3, and HPAE-A1, A3), despite increased cytotoxicity (Figure S7). These results demonstrate that the new optimization strategy of removing small MW components is applicable to HPAE groups with different MWs for enhanced gene delivery. In addition, the MW has a significant effect on the transfection performance of the optimized HPAEs as a *M*_w,GPC_ around 24 kDa and 28 kDa is more favorable for enhanced transfection efficiency with RDEBK cells and HEK cells, respectively.

**Figure 5:**
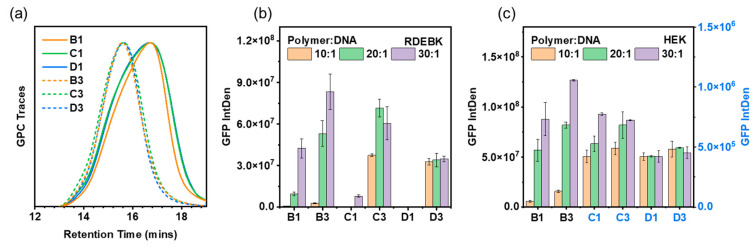
GPC characterization and in vitro assessment to investigate the applicability of removing small MWs components in HPAEs with different branching ratios (HPAE-B1, B3, HPAE-C1, C3, and HPAE-D1, D3). (**a**) GPC traces of HPAE-B1, HPAE-C1, HPAE-D1, and their optimized products HPAE-B3, HPAE-C3, HPAE-D3; transfection efficiency of polyplexes formulated with HPAE-B1, B3, HPAE-C1, C3, and HPAE-D1, D3 at different *w/w* ratios 48 h post-treatment: GFP expression of (**b**) RDEBK and (**c**) HEK cells treated with HPAE/DNA polyplexes. GFP expression of C1, C3, D1 and D3 correspond to the coordinate axis on the right (blue) due to their low intensity.

The branched structure is another crucial structural parameter determining HPAE transfection efficiency and safety. Previous structure−property relationship studies indicate that a proper BD in HPAEs can improve gene transfection efficiency by facilitating the DNA binding and formulation of smaller polyplexes with a higher surface charge [34,35]. However, the optimal BD of HPAEs might vary due to the loss of small *M*_w_ components in the optimization. To validate the above component optimization strategy in HPAE groups with different BDs, HPAE-C1 and HPAE-D1, which have different BDs from HPAE-B1, were prepared by varying the feeding ratios of PTTA to BDA (0.1:1 for HPAE-B1, 0.2:1 for HPAE-C1, 0.3:1 for HPAE-D1, molar ratios in Table S1). Consequently, HPAE-B1, HPAE-C1, and HPAE-D1 were synthesized with similar MWs (*M*_w,GPC_ of 12.6 kDa, 13.5 kDa, and 12.8 kDa, Figure 5a, entry 5, entry 7, and entry 9 in Table 1) and different BDs—0.1 for HPAE-B1, 0.2 for HPAE-C1, and 0.3 for HPAE-D1, respectively (Figure S3, Figure S8, Figure S9, and Figure S11). The Mark–Houwink (MH) plotted alpha values for HPAE-B1, C1, and D1 of 0.34, 0.31, and 0.28, respectively (Figure S10), which further proved the enhanced branched structure from HPAE-B1 to HPAE-D1. Then, HPAE-B1, HPAE-C1, and HPAE-D1 were applied to the three-step precipitation to generate their optimized products of HPAE-B3, HPAE-C3, and HPAE-D3. After removing small MW components, the MWs of these optimized HPAEs were kept similar (*M*_w,GPC_ around 21 kDa, Figure 5a, entry 6, entry 8, and entry 10 in Table 1). The BDs of the optimized HPAEs slightly increased (BD of 0.13, 0.23, and 0.35 for HPAE-B3, C3, and D3, respectively) compared to their original products, which was confirmed by ^1^H NMR (Figure S11).

Figure 5b,c outlines the HPAE gene delivery efficacy in the RDEBK and HEK cells after transfection. As expected, the optimized HPAE-B3, HPAE-C3, and HPAE-D3 exhibited significant GFP expression enhancement compared to their original counterparts (HPAE-B1, HPAE-C1, and HPAE-D1). Particularly for RDEBK transfection, it can be found that although HPAE-C1 and HPAE-D1 with higher BDs (0.2 for HPAE-C1 and 0.3 for HPAE-D1) showed limited gene transfection efficacy at all *w/w* ratios, after removing the small MW components, their GFP expression were orders-of-magnitude enhanced under the same transfection conditions (Figure 5b) with minimal cytotoxicity (>90%, Figure S12) at all *w/w* ratios. In addition, for both the RDEBK and HEK cell transfection, the optimized HPAE-B3 with the lowest BD (~0.13) exhibited the highest transfection efficacy (Figure 5b,c) with no apparent cytotoxicity (Figures S12 and S13). This is consistent with the structure−activity relationship of original HPAEs, that is, HPAE-B1 with the lowest BD exhibited the best transfection performance among HPAE-B1, C1, and D1. These results again prove the universality of the proposed HPAE optimization strategy of removing small MW components from HPAE. This strategy can be successfully applied to enhance the transfection performance of HPAEs with different BDs while preserving high cell viability in both RDEBK and HEK cells.

## 4. Conclusion

In this work, a small MW component elimination strategy was proposed for the first time to enhance the gene transfection efficacy of HPAEs. A series of HPAEs with different polymer component combinations were achieved by a stepwise precipitation method. Through systematically evaluating their transfection behavior in vitro, it was demonstrated that removing small MW components in HPAEs could significantly promote the transfection efficacy of HPAE. This optimization strategy was also validated in HPAEs with different MWs and BDs. This work provides new insights into the understanding of the HPAE structure–property relationship, which will facilitate the development of high-efficiency HPAE gene delivery vectors in the future.

## Figures and Tables

**Table 1:** The GPC characterization results of the HPAE polymers ^1^.

Entry	Polymer	*M*_n,GPC_ (kDa)	*M*_w,GPC_ (kDa)	*Đ*
1	HPAE-A1	4.8	15.0	3.1
2	HPAE-A2	6.5	17.6	2.7
3	HPAE-A3	9.9	23.6	2.4
4	HPAE-A4	17.4	34.9	2.0
5	HPAE-B1	5.3	12.6	2.4
6	HPAE-B3	10.5	21.0	2.0
7	HPAE-C1	5.0	13.5	2.7
8	HPAE-C3	10.5	21.7	2.0
9	HPAE-D1	5.0	12.8	2.6
10	HPAE-D3	11.1	20.7	1.8
11	HPAE-E1	5.7	20.1	3.5
12	HPAE-E3	8.7	28.3	3.2

^1^ Determined by the GPC RI detector.

## Data Availability

The data presented in this study are available on request from the corresponding author.

## Supplementary Materials

The following supporting information can be downloaded at: https://www.mdpi.com/article/10.3390/polym15061518/s1, Figure S1: GPC traces of HPAE-A1 sampled at different polymerization stages, Figure S2: Cell viability of RDEBK cells 48 h post transfection by HPAE- A1, A2, A3, and A4, Figure S3: GPC traces of HPAE-B1 sampled at different polymerization stages, Figure S4: GPC traces of HPAE-E1 sampled at different polymerization stages, Figure S5: ^1^H NMR spectra of HPAE-E1. Figure S6: Cell viability of RDEBK cells 48 h post transfection by HPAE-B1, B3, A1, A3, E1, and E3, Figure S7: Cell viability of HEK cells 48 h post transfection by HPAE-B1, B3, A1, A3, E1, and E3, Figure S8: GPC traces of HPAE-C1 sampled at different polymerization stages, Figure S9: GPC traces of HPAE-D1 sampled at different polymerization stages, Figure S10: Mark–Houwink plots of HPAE-B1, C1, D1, B3, C3, and D3, Figure S11: ^1^H NMR spectra of HPAE-B1, C1, D1, B3, C3, and D3, Figure S12: Cell viability of RDEBK cells 48 h post transfection by HPAE-B1, B3, C1, C3, D1, and D3, Figure S13: Cell viability of HEK cells 48 h post transfection by HPAE-B1, B3, C1, C3, D1, and D3; Table S1: Monomer molar ratios and reaction conditions for HPAE synthesis.
