# Supplementary material for: A New Optimization Strategy of Highly Branched Poly(β-Amino Ester) for Enhanced Gene Delivery: Removal of Small Molecular Weight Components
Source: Polymers (Basel). 2023 Mar 18;15(6):1518. doi: 10.3390/polym15061518 (PMC10051207; doi:10.3390/polym15061518)
Supplement: Supplementary file 1 [file polymers-15-01518-s001.zip › polymers-2265030-supplementary.pdf]

Supporting Information

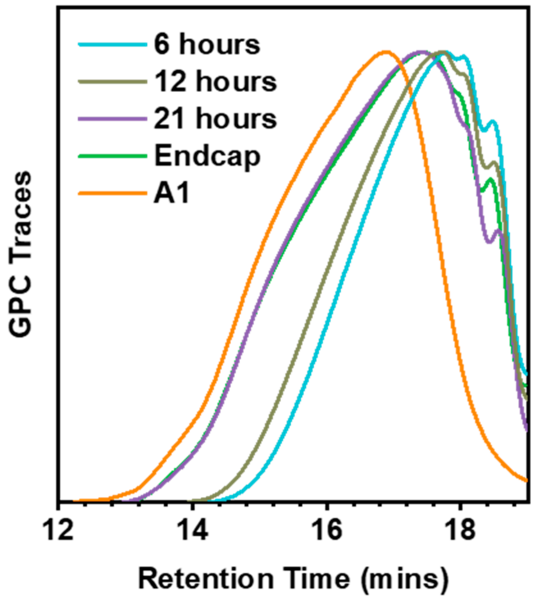

Figure S1. GPC traces of HPAE-A1 sampled at different polymerization stages.

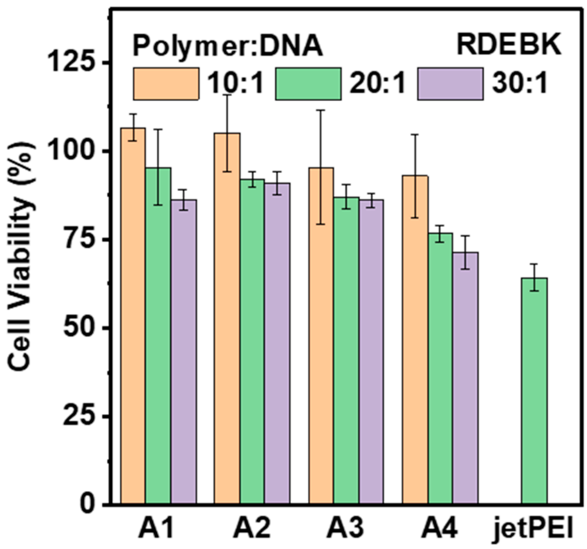

Figure S2. Cell viability of RDEBK cells 48 h post transfection by HPAE-A1 to A4.

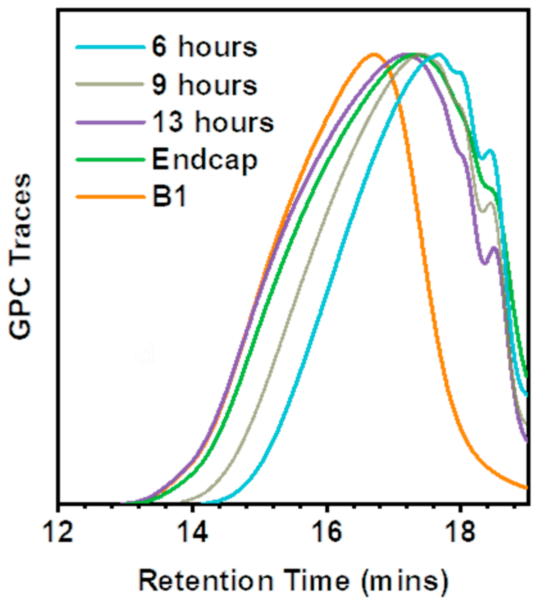

Figure S3. GPC traces of HPAE-B1 sampled at different polymerization stages.

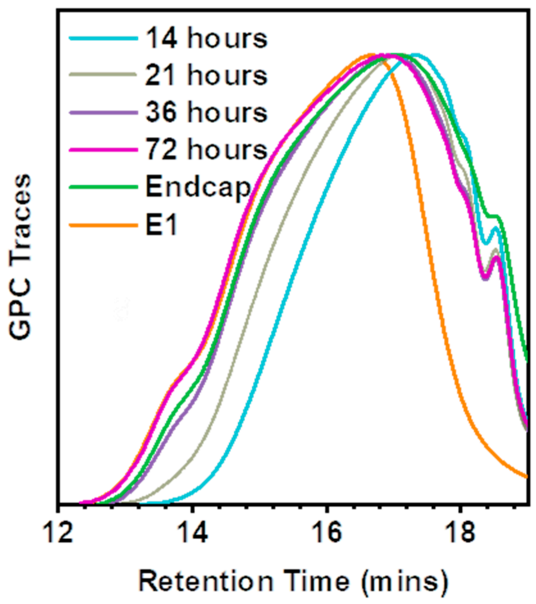

Figure S4. GPC traces of HPAE-E1 sampled at different polymerization stages.

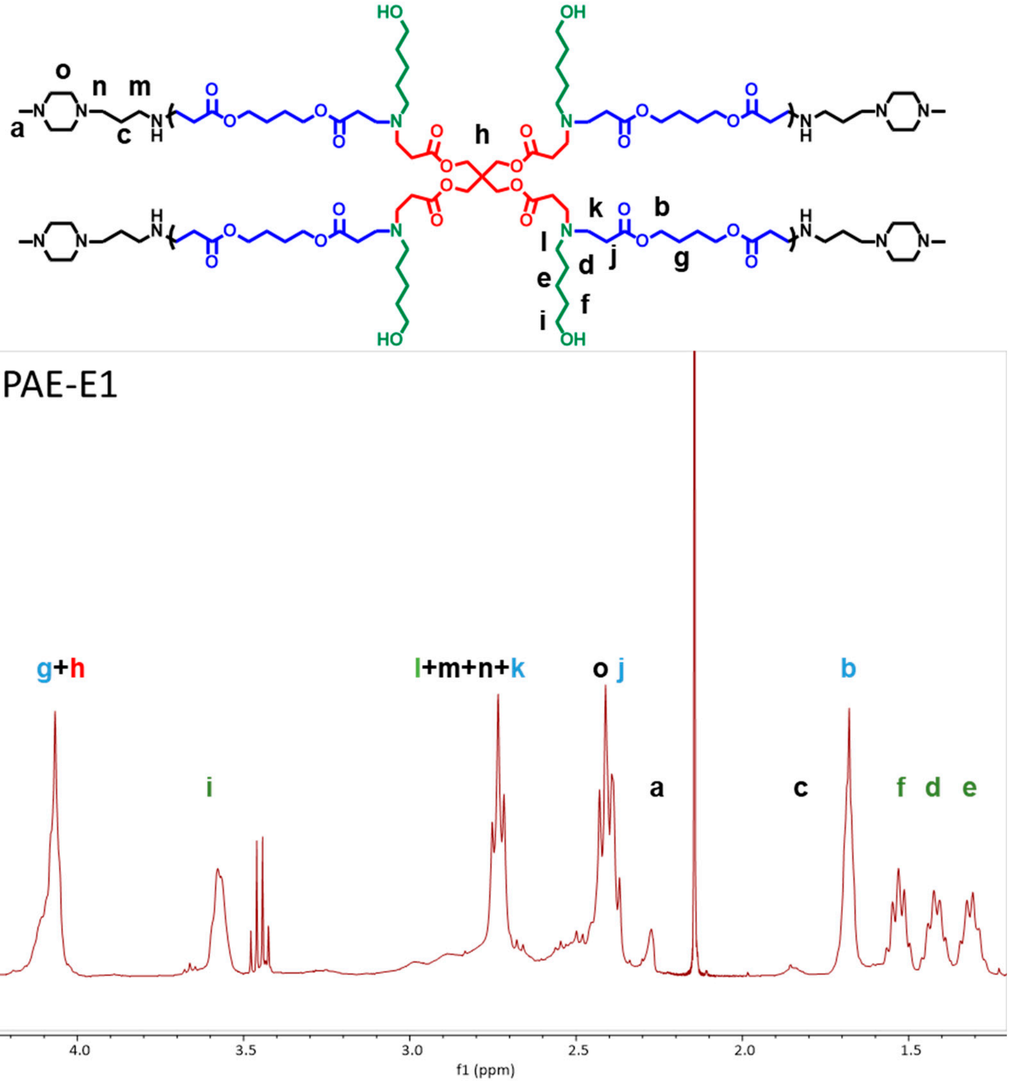

Figure S5. ^{1}H NMR spectra of HPAE-E1. Branching degree is 0.13, which is calculated from equation: the molar ratio of PTTA/BDA= $[(I_{g+h} - I_b) / 8] / [I_b / 4]$, where I_{g+h}, and I_b stand for the integral intensity of peak g+h, and b in ^{1}H NMR spectra.

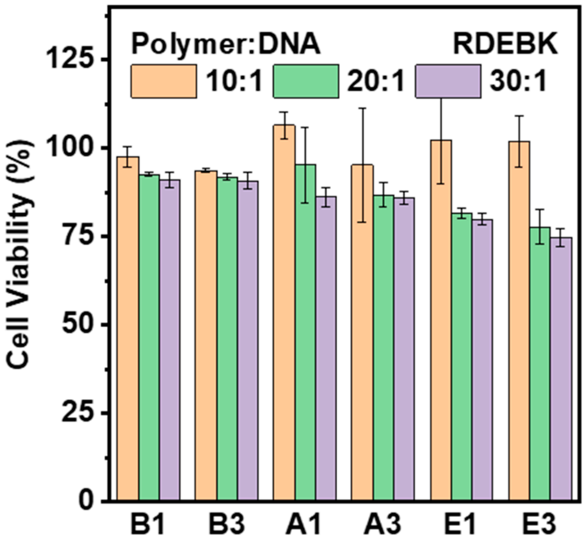

Figure S6. Cell viability of RDEBK cells 48 h post transfection by HPAE-B1, B3, A1, A3, E1, and E3.

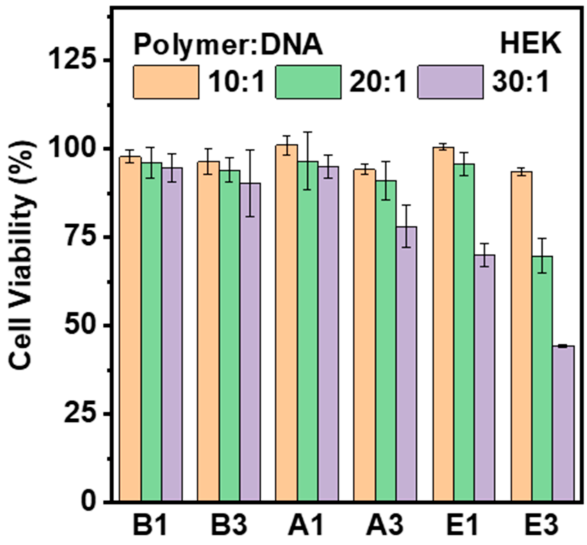

Figure S7. Cell viability of HEK cells 48 h post transfection by HPAE-B1, B3, A1, A3, E1, and E3.

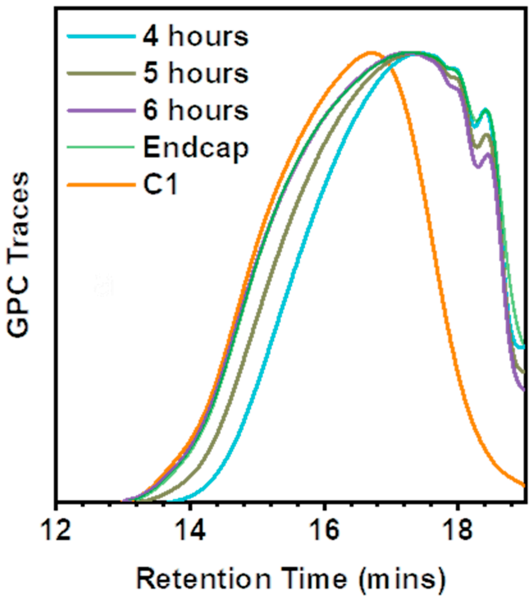

Figure S8. GPC traces of HPAE-C1 sampled at different polymerization stages.

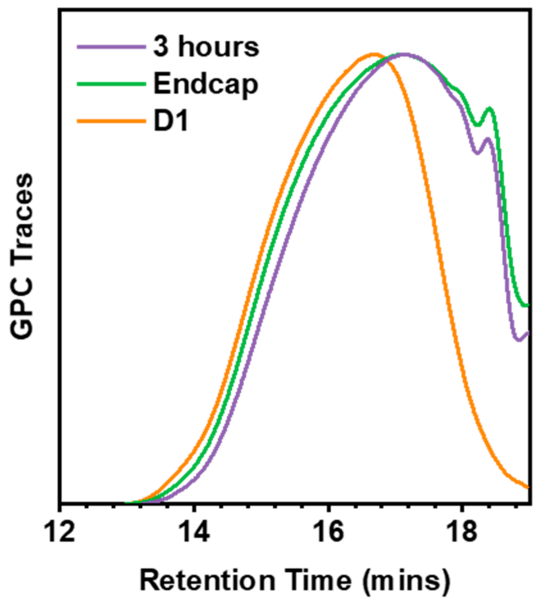

Figure S9. GPC traces of HPAE-D1 sampled at different polymerization stages.

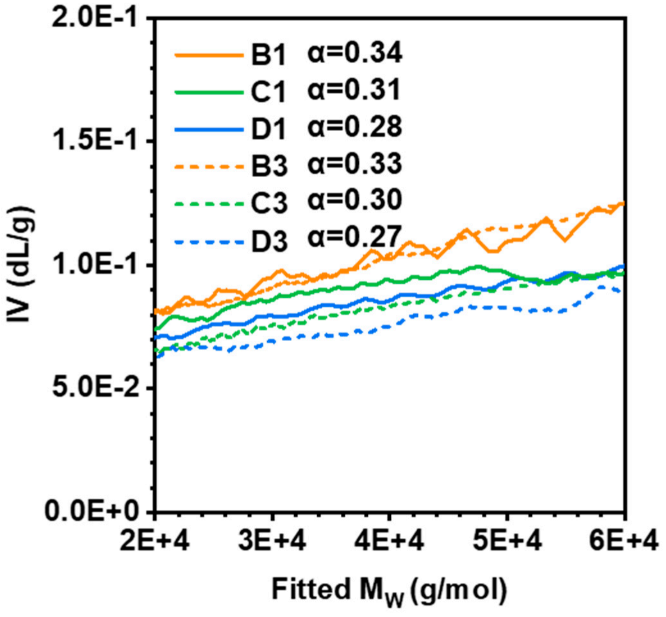

Figure S10. Mark-Houwink plots of HPAE-B1, C1, D1, B3, C3, and D3.

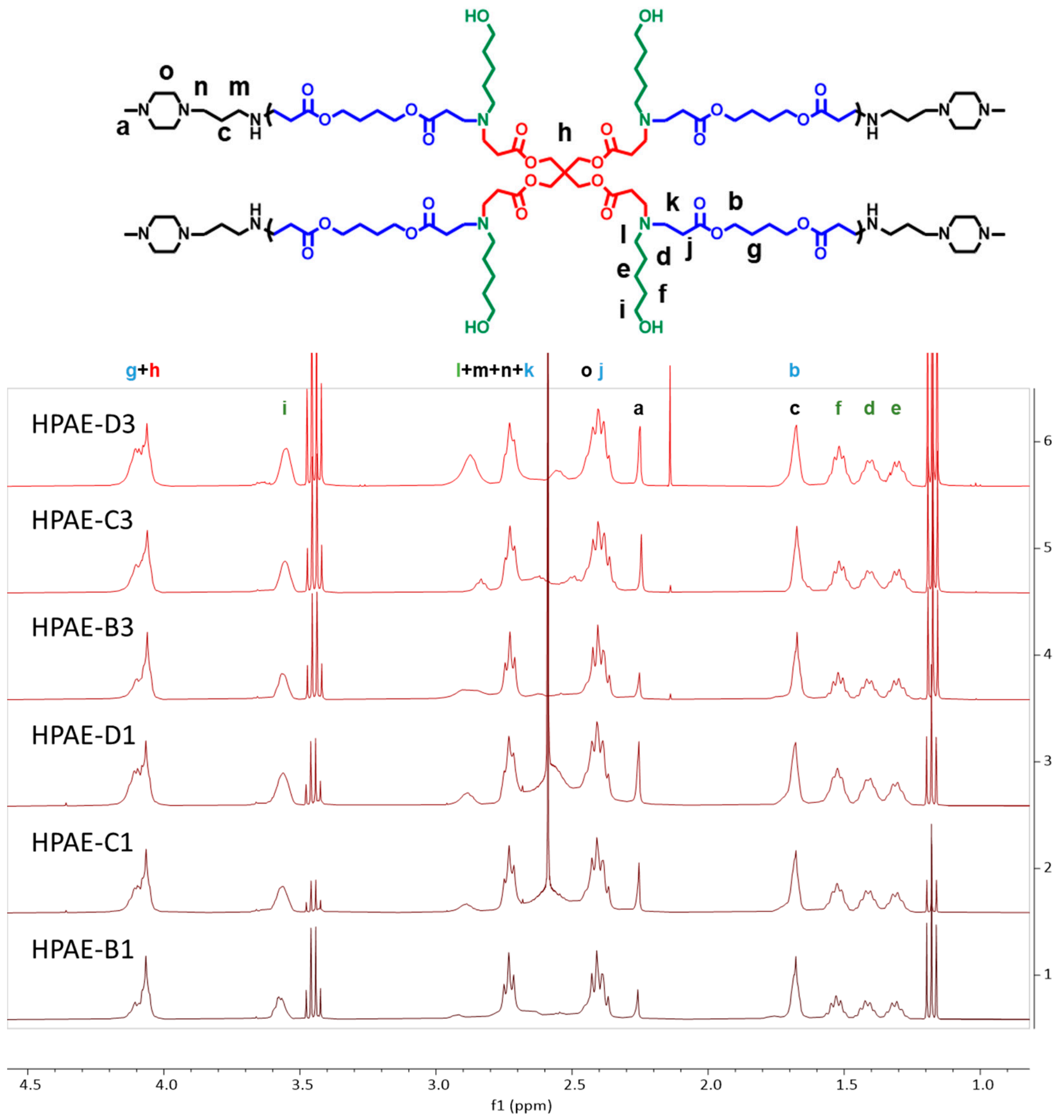

Figure S11. ^{1}H NMR spectra of HPAE-B1, C1, D1, B3, C3, and D3. Branching degrees of HPAEs are calculated from equation: the molar ratio of PTTA/BDA= $[(I_{g+h} - I_b) / 8] / [I_b / 4]$, where I_{g+h}, and I_b stand for the integral intensity of peak g+h, and b in ^{1}H NMR spectra.

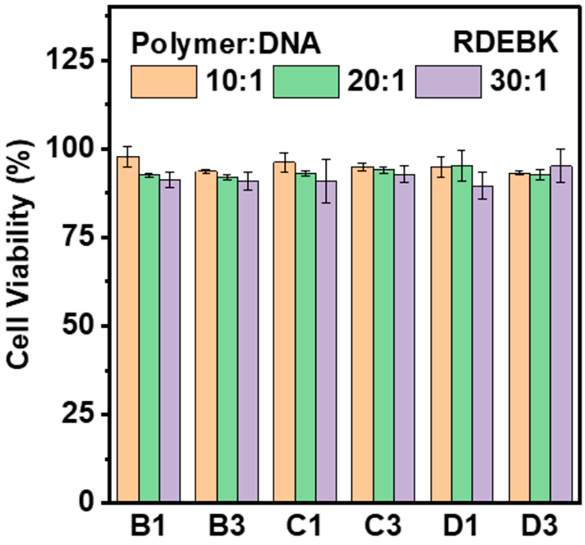

Figure S12. Cell viability of RDEBK cells 48 h post transfection by HPAE-B1, B3, C1, C3, D1, and D3.

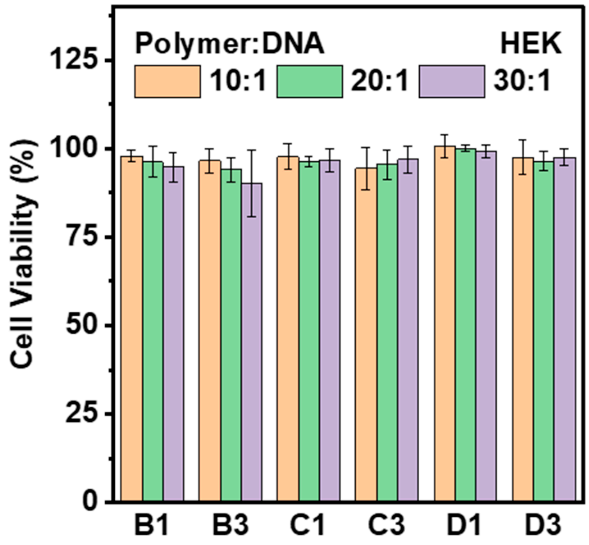

Figure S13. Cell viability of HEK cells 48 h post transfection by HPAE-B1, B3, C1, C3, D1, and D3.

Table S1. Monomer feeding ratios and reaction conditions for HPAE synthesis.

	Monomer molar ratio				Reaction condition		
HPAE	BDA	PTTA	S5	E7	Temperature	Solvent	Concentration
A1[1]	1	0.1	1	0.8	90 °C	DMSO	30% w/v
B1[1]	1	0.1	1	0.8	90 °C	DMSO	30% w/v
C1	1	0.2	0.16	0.9	90 °C	DMSO	30% w/v
D1	1	0.3	0.13	1.0	90 °C	DMSO	30% w/v
E1[1]	1	0.1	1	0.8	90 °C	DMSO	30% w/v

[1] The polymerizations were stopped at different times – 21 h for HAPE-A1, 13 h for HPAE-B1 and 72 h for HPAE-E1.
